# A probabilistic framework to predict protein function from interaction data integrated with semantic knowledge

**DOI:** 10.1186/1471-2105-9-382

**Published:** 2008-09-18

**Authors:** Young-Rae Cho, Lei Shi, Murali Ramanathan, Aidong Zhang

**Affiliations:** 1 Department of Computer Science and Engineering, State University of New York, Buffalo, NY, USA; 2 Department of Pharmaceutical Sciences, State University of New York, Buffalo, NY, USA

## Abstract

**Background:**

The functional characterization of newly discovered proteins has been a challenge in the post-genomic era. Protein-protein interactions provide insights into the functional analysis because the function of unknown proteins can be postulated on the basis of their interaction evidence with known proteins. The protein-protein interaction data sets have been enriched by high-throughput experimental methods. However, the functional analysis using the interaction data has a limitation in accuracy because of the presence of the false positive data experimentally generated and the interactions that are a lack of functional linkage.

**Results:**

Protein-protein interaction data can be integrated with the functional knowledge existing in the Gene Ontology (GO) database. We apply similarity measures to assess the functional similarity between interacting proteins. We present a probabilistic framework for predicting functions of unknown proteins based on the functional similarity. We use the leave-one-out cross validation to compare the performance. The experimental results demonstrate that our algorithm performs better than other competing methods in terms of prediction accuracy. In particular, it handles the high false positive rates of current interaction data well.

**Conclusion:**

The experimentally determined protein-protein interactions are erroneous to uncover the functional associations among proteins. The performance of function prediction for uncharacterized proteins can be enhanced by the integration of multiple data sources available.

## Background

Since the completion of sequencing the human genome [1], discovering the underlying principles of interactions and the functional roles of proteins has been in the spotlight in the post-genomic era. The functional characterization of newly determined proteins has become one of the most crucial challenges. The initial efforts have been carried out by sequential or structural homology searches using computational algorithms such as FASTA [2] and BLAST [3], and the tasks of predicting protein function are still progressing with a massive amount of data [4,5].

The availability of complete genomes in a wide variety of organisms has shifted the single-gene-based function prediction problem to a genome-level. Several approaches have been introduced on the basis of correlated evolution mechanisms of genes. The conserved gene neighborhood across different, distantly related genomes reveals the potential functional linkages [6]. The domain fusion analysis infers a pair of proteins that interact with each other and thus perform a related function [7]. The phylogenetic profiles represent the pattern of presence or absence of a protein in a set of organisms. Two proteins are considered to be functionally linked if they have the same phylogenetic profiles [8].

In recent years, the data generated by high-throughput techniques have facilitated the functional classification. For example, microarrays monitor the expression levels of thousands of genes, and the correlated expression profiles of the genes can be interpreted as their functional relatedness [9]. Protein-protein interaction data, enriched by high-throughput experiments including yeast two-hybrid systems [10] and mass spectrometry [11], have provided the important clues of functional associations between proteins. The integrated protein interaction networks have been built from the heterogeneous interaction data sources. Accordingly, numerous computational methods have been supplemented for uncovering the functional information of uncharacterized proteins in the networks.

In this study, we explore an effective methodology for predicting functions of unknown proteins using the connectivity of protein interaction networks. Previously, a number of approaches have been proposed to predict protein function from protein interaction networks. The neighbor counting method [12], which is also called the majority-rule based method, searches the most common function among the neighbors, i.e., interacting partners, of an unknown protein. The commonality of the functions of neighbors can be statistically evaluated. Hishigaki et al. [13] used a chi-square formula to calculate the statistical significance of the functions of neighbors. Because the neighbor counting and related methods focus on the direct neighborhood, they are problematic if an unknown protein has a small number of interacting partners annotated or a large number of false positive interactions.

Several graph theoretic approaches took into account the global topology of protein interaction networks. Vazquez et al. [14] and Karaoz et al. [15] attempted to maximize the functional consistency through neighboring in the whole network. Nabieva et al. [16] applied the concept of functional flow that is propagated from an annotated protein to unannotated proteins. Probabilistic approaches have also been suggested for function prediction. Deng et al. [17] introduced a statistical framework using the Markov Random Field (MRF) model in a Gibbs distribution. They used a quasi-likelihood approach to estimate the parameters in the MRF model. Lee et al. [18] developed a kernel logistic regression (KLR) method based on diffusion kernels and incorporated all indirect neighborhoods in the networks. Chua et al. [19] also considered the indirect neighbors with length-2. They computed the functional similarity score between two proteins, which was derived from the symmetric difference of neighbors and the reliability of the data sources used. Kirac et al. [20] used the annotation patterns in the neighborhood for the function prediction. They calculated the similarity between the annotation pattern of an unknown protein and the set of annotation patterns for each function.

Most of the previous approaches to predict protein function from protein interaction networks are based on the assumption that two interacting proteins have a similar function or share functions. However, these tasks overestimate the tendency of functional links between the proteins that interact with each other. In addition, it is recognized that current interaction data include a substantial number of false positives and false negatives although they have been curated using various computational techniques. It signifies that the high-throughput experimental methods frequently generate spurious data, and the set of interactions accumulated in a large scale is still incomplete. Because of the inappropriateness of direct use of current interaction data for function prediction, the integration of functional knowledge elicited from other biological resources is necessary.

In this article, we propose a novel probabilistic approach for function prediction from protein interaction networks. We integrate the connectivity of protein interaction networks with the semantic knowledge in the Gene Ontology (GO) database for the purpose of improving the accuracy of function prediction. First, we measure the functional similarity between interacting proteins using GO. Next, we present a probabilistic framework to compute the confidence in function prediction for each unknown protein. Our experimental results show that this approach is robust to current erroneous data and thus predicts function more accurately than previous methods.

### Integration of interactions with semantic knowledge

Semantic knowledge, which is also called ontology, is the representation in which concepts are described by their meanings and their relationships to each other [21]. It is typically represented as a hierarchical tree structure or a directed acyclic graph (DAG). The Gene Ontology (GO) [22] is currently one of the most widely used databases that provide the semantic knowledge related to biological processes and molecular functions. Because the GO terms and their annotations are continually updated as research progresses, they might include errors. However, we employ the GO for the integration with protein-protein interactions because of its comprehensiveness. The relationships between GO terms and the annotations can be exploited to measure the functional similarity between interacting proteins.

Recently, various measurements of functional similarity between proteins using GO have been proposed [23-28]. Such measures can be classified into two distinct categories: structure-based approaches and annotation-based approaches. Suppose each protein is annotated on the most specific GO terms on the paths from the root term. Structure-based approaches utilize the path length or the common parent terms between two GO terms, on which each interacting protein is annotated. Two interacting proteins are functionally more similar if two GO terms whose annotations include each of them are closer each other, or the GO terms have a larger number of common parent terms in the GO structure. As a combination of the two factors, the path length from the root to the most specific common parent term can be taken into consideration. (See Methods for details of structure-based approaches.) However, as a weakness, they depend on the critical assumption that all the edges in the structure represent the same specificity.

Annotation-based approaches focus on the number of proteins annotated on GO terms. They suppose the GO annotations follow the transitivity property, i.e., if a protein is annotated on a term *T*, then it is also annotated on more general terms on the paths from *T *to the root in the GO structure. The set of proteins annotated on the root term becomes transitive. Under this property, the number of annotated proteins on a term *T *is capable of quantifying the specificity of *T*. The functional similarity between interacting proteins is then measured based on the commonality of the annotations of two terms on which they are annotated. The more annotations two terms share, the more similar they are. The measured similarity can be also normalized by the number of annotated proteins on the most specific individual terms whose annotations include each interacting protein. (See Methods for details of annotation-based approaches.) Annotation-based approaches generally perform better than structure-based approaches when the annotations in GO are fairly complete.

### Function prediction algorithm

Our approach predicts multiple functions for each protein, which is functionally uncharacterized but has the evidence of interactions. It is based on the Bayesian formula using the functional similarity measured by the metrics described above. Suppose protein-protein interaction data contain a set of *n *distinct proteins, P = {*p*_1_,...,*p*_*n*_}. In P, *p*_1_,...,*p*_*k *_(*k *<*n*) are functionally annotated and *p*_*k*+1_,...,*p*_*n *_are unannotated. We predict functions of an unannotated protein *p*_*i *_where *k *<*i *≤ *n*. Let $P_{f}=\{p_{f_{1}},...,p_{f_{m}}\}$ be the set of proteins annotated on a function *f*, and $R_{f_{1}},...,R_{f_{m}}$ be the functional similarity between interacting proteins, *p*_*i *_and $p_{f_{j}}$ where 1 ≤ *j *≤ *m*. If there is no interaction evidence between *p*_*i *_and $p_{f_{j}}$, then the functional similarity $R_{f_{j}}$ becomes 0. According to the Bayes theorem, the conditional probability that *p*_*i *_has the function *f *given $R_{f_{1}},...,R_{f_{m}}$ is defined as:

(1) $$P(f=1|R_{f_{1}},...,R_{f_{m}})=\frac{P(R_{f_{1}},...,R_{f_{m}}|f=1)P(f=1)}{P(R_{f_{1}},...,R_{f_{m}})},$$

where *P*(*f *= 1) is the prior probability that *p*_*i *_has the function *f*, *P *($R_{f_{1}},...,R_{f_{m}}$) is the probability that *p*_*i *_interacts with $p_{f_{1}},...,p_{f_{m}}$ having the functional similarity of $R_{f_{1}},...,R_{f_{m}}$, and *P*($R_{f_{1}},...,R_{f_{m}}$|*f *= 1) is the conditional probability that *p*_*i *_interacts with $p_{f_{1}},...,p_{f_{m}}$ having the functional similarity of $R_{f_{1}},...,R_{f_{m}}$ given that *p*_*i*_has the function *f*. Based on the assumption that the events of the interactions between *p*_*i *_and $p_{f_{1}},...,p_{f_{m}}$ independently occur,

(2) $$P(R_{f_{1}},...,R_{f_{m}}|f=1)=\prod_{j=1}^{m}P(R_{f_{j}}|f=1).$$

Equation 1 is then transformed into

(3) $$\begin{array}{l} P(f=1|R_{f_{1}},...,R_{f_{m}})=\\ \frac{\prod_{j=1}^{m}P(R_{f_{j}}|f=1)P(f=1)}{\prod_{j=1}^{m}P(R_{f_{j}}|f=1)P(f=1)+\prod_{j=1}^{m}P(R_{f_{j}}|f=0)P(f=0)}, \end{array}$$

where *P*(*f *= 0) is the probability that *p*_*i *_does not have the function *f*.

Let *M*_*f *_be the maximum functional similarity. The allowable amount in functional similarity for *p*_*i *_and $p_{f_{j}}$ to have the same function *f *can be *R*_*f *_= *M*_*f *_- $R_{f_{j}}$. We assume *P*($R_{f_{j}}$|*f *= 1) follows a binomial distribution.

(4) $$P(R_{f_{j}}|f=1)=\left(\begin{array}{c} M_{f}\\ R_{f} \end{array}\right)P_{f}^{R_{f}}{\left(1-P_{f}\right)}^{M_{f}-R_{f}},$$

where *P*_*f *_is the probability that two proteins have the same function *f*. We can approximate the binomial distribution to a normal distribution with the mean *μ *and variance *σ*^2^.

(5) $$P(R_{f_{j}}|f=1)=\frac{1}{\sqrt{2\pi }\sigma_{f+}}e^{-\frac{{\left(R_{f_{j}}-\mu_{f+}\right)}^{2}}{\sigma_{f+}^{2}}}.$$

In the same way,

(6) $$P(R_{f_{j}}|f=0)=\frac{1}{\sqrt{2\pi }\sigma_{f-}}e^{-\frac{{\left(R_{f_{j}}-\mu_{f-}\right)}^{2}}{\sigma_{f-}^{2}}}.$$

Then, Equation 3 can be re-written as

(7) $$P(f=1|R_{f_{1}},...,R_{f_{m}})=\frac{\lambda_{f}}{\lambda_{f}+1},$$

where

(8) $$\lambda_{f}=\frac{\sigma_{f-}^{m}}{\sigma_{f+}^{m}}\cdot e^{-\sum_{j=1}^{m}\left(\frac{{\left(R_{f_{j}}-\mu_{f+}\right)}^{2}}{\sigma_{f+}^{2}}-\frac{{\left(R_{f_{j}}-\mu_{f-}\right)}^{2}}{\sigma_{f-}^{2}}\right)}\cdot \frac{P(f=1)}{P(f=0)}.$$

*μ*_*f*+ _and $\sigma_{f+}^{2}$ are calculated by the functional similarity between *p*_*i *_and the proteins annotated on *f*. Similarly, *μ*_*f*- _and $\sigma_{f-}^{2}$ are calculated by the functional similarity between *p*_*i *_and the proteins that are not in the annotation of *f*. *P*(*f *= 1) becomes the ratio of the number of proteins having *f *to the total number of known proteins, and *P*(*f *= 0) is 1 - *P*(*f *= 1). As an alternative to Formula 7, we compute log(*λ*_*f*_), which can be the prediction conifdence for *p*_*i *_to the function *f*.

## Results

### Functional linkage of protein-protein interactions

We assessed the tendency of functional linkage between interacting proteins in current interaction databases. We extracted the interaction data of Saccharomyces cerevisiae from three databases: 12352 interactions from MIPS [29], 17186 from DIP [30] and 56860 from BioGRID [31]. We then computed the functional similarity between interacting proteins using two selected similarity measures: a structure-based method and an annotation-based method. The functional hierarchy and annotations from FunCat [32] in MIPS were used for the similarity measures. We also calculated the functional consistency using the Jaccard index.

**Figure 1:**
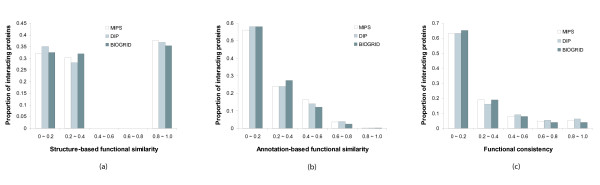
**Distribution of interacting proteins with respect to the (a) structure-based functional similarity, (b) annotation-based functional similarity and (c) functional consistency**. The interaction data from the MIPS, DIP and BioGRID databases were used. The functional categories and annotations were obtained from FunCat in MIPS. (a) The functional similarity of each interacting protein pair was measured by the maximum structure-based similarity of the pair-wise functions they have in a hierarchy. More than 60% of interacting pairs have the functional similarity less than 0.4. (b) The functional similarity of each interacting protein pair was also measured by the maximum annotation-based similarity of the pair-wise functions they have. Around 60% of interacting pairs have the functional similarity less than 0.2. (c) The functional consistency of each interacting protein pair was finally measured by the proportion of the common functions they share. As similar to the distribution in (b), more than 60% of interacting pairs have the functional consistency less than 0.2.

First, we measured the functional similarity of each interacting protein pair using the structure-based method in Formula 13. Figure 1(a) shows the distribution of interacting protein pairs with respect to their structure-based functional similarity. Importantly, only 38% of the interacting pairs in MIPS, 37% in DIP and 35% in BioGRID have the functional similarity of greater than 0.8. The other interacting pairs in the databases have very low rates of similarity which are less than 0.4. Moreover, more than 30% of the interacting pairs have the functional similarity of 0, i.e., they do not have any common functions. It is interesting that there are no interacting pairs with the functional similarity in the range between 0.4 and 0.8. The result indicates that more than 60% of the interactions in the databases do not perform any similar functions.

Next, we measured the functional similarity of each interacting protein pair using the annotation-based method in Formula 14. Figure 1(b) shows the distribution of interacting protein pairs with respect to their annotation-based functional similarity. A very few interacting pairs in the three databases are co-annotated on specific functional categories. Furthermore, a large fraction of the interacting pairs, 56% in MIPS and 58% in DIP and BioGRID, have the similarity less than 0.2, i.e., they rarely have common annotations. It indicates that around 60% of the interacting proteins in the databases are not co-annotated on any specific functions.

Finally, we observed the functional consistency of each interacting protein pair using the Jaccard index. According to the transitivity property of functional annotations, if a protein is annotated on a function, then it also has more general functions on the paths towards the root in the hierarchical structure. We calculated the number of common functions among the number of all distinct functions of an interacting protein pair. Figure 1(c) shows the distribution of interacting protein pairs with respect to their functional consistency. The overall distributing pattern is similar to that in Figure 1(b). Only 18% of the interacting pairs in MIPS, 21% in DIP and 16% in BioGRID have the consistency of greater than or equal to 0.4. On the other hand, 63% in MIPS and DIP and 65% in BioGRID have the consistency of less than 0.2, and their common functions are likely to be very general ones, which are located on the upper levels in the functional hierarchy. This result thus implies that more than 60% of the interacting proteins in the databases do not share any specific functions.

### Comparison of functional similarity measurements

We evaluated the effectiveness of the functional similarity measurements to choose the best one for our function prediction approach. We used the full version of protein-protein interaction data of Saccharomyces cerevisiae from DIP [30], which includes 4928 distinct proteins and 17186 interactions. The GO terms and their annotations were downloaded from the Gene Ontology database [22]. Upon selecting the GO terms that have the annotation of two or more proteins in biological process and molecular function categories, we obtained total 2456 GO terms. In both categories, the root terms have the annotations of 5871 proteins. As a structure-based similarity measurement, we chose Formula 13, which is the most advanced metric in the category. As an annotation-based similarity measurement, we used Formula 14 because a previous study [24] has shown that it has the best performance. We compared the functional similarity of interacting proteins to their interaction reliability that is measured based on connectivity [33].

**Figure 2:**
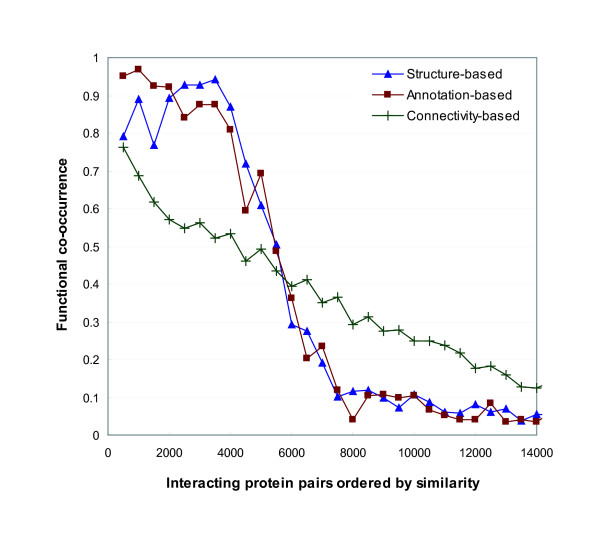
**Functional co-occurrence rates of interacting protein pairs sorted by their similarity in a descending order**. We compared three similarity measurements by functional co-occurrence. The interaction data from DIP were used. For each interacting protein pair, we measured the structure-based similarity in Formula 13, the annotation-based similarity in Formula 14 and connectivity-based interaction reliability in [33]. For the structure-based and annotation-based similarity, we used the GO terms and annotations in Biological Process and Molecular Function categories. We then sorted the pairs by their similarity in a descending order, and calculated the average functional co-occurrence rates for every 500 pairs. That is, we inspected how many pairs among 500 pairs co-occurred in the same functional categories from FunCat in MIPS. The interacting pairs with high structure-based and annotation-based similarity have higher rates of functional co-occurrence than those with high connectivity-based reliability. Moreover, in the range of top 4000 pairs, the annotation-based similarity performs better than the structure-based similarity.

We first evaluated interacting pairs by functional co-occurrence in the categories from FunCat [32] in MIPS. We sorted the pairs in a descending order by similarity. We then grouped every 500 interacting pairs in the order and calculated the fraction of the pairs, which are co-occurred in the same functional categories, for each group. Figure 2 shows the alteration of the functional co-occurrence rates. The first several groups have very high co-occurrence rates, but the rates substantially decrease after the 8th group. As expected from Figure 1, the interacting pairs after the 7000th, which correspond to around 60% of total pairs, remain in low co-occurrence rates. When we consider the first eight groups, the annotation-based similarity showed better performance than the structure-based similarity. Using the structure-based similarity, the 5th, 6th and 7th groups have higher co-occurrence rates rather than the first four groups. The structure-based and annotation-based functional similarity outperformed the connectivity-based interaction reliability. The interacting protein pairs with high similarity have higher functional co-occurrence rates, and those with low similarity have lower rates.

**Figure 3:**
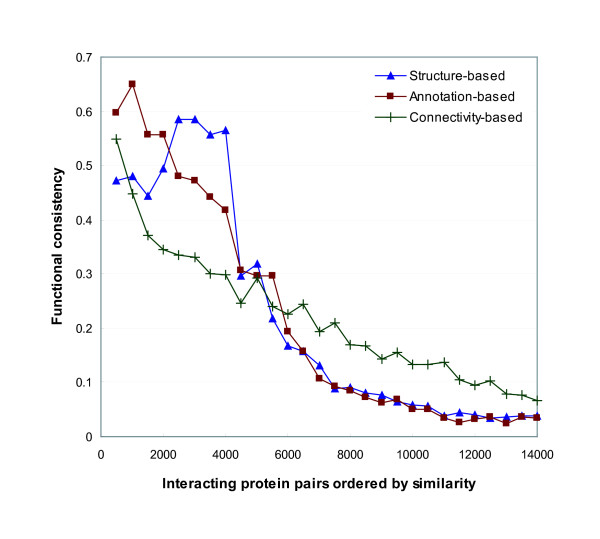
**Functional consistency of interacting protein pairs sorted by their similarity in a descending order**. We compared the same similarity measurements to those in Figure 2 by functional consistency. Using the sorted interacting pairs by their similarity in a descending order, we calculated the average functional consistency for every 500 pairs. The functional consistency of a pair is computed by the ratio of the number of common functions to the number of all distinct functions that the two proteins have. The general pattern of functional consistency is similar to that of functional co-occurrence in Figure 2. When we use the annotation-based similarity, the functional consistency monotonically decreases as the similarity of interacting pairs declines.

Next, we evaluated the ordered interacting pairs by functional consistency. Similar to the calculation in the previous section, the functional consistency was computed by the ratio of the number of common functions to the number of all distinct functions of an interacting protein pair. Figure 3 shows the alteration of the average functional consistency of every 500 pairs. The pattern of functional consistency with respect to similarity is comparable to that of functional co-occurrence in Figure 2. For the first four groups, the annotation-based similarity measure showed the higher consistency than the structure-based similarity and the connectivity-based interaction reliability. For the groups after the 7000th pair, the functional consistency is lower than 0.1. As a result from Figure 2 and 3, the annotation-based method in Formula 14 performs better than the others for measuring the functional similarity between interacting proteins.

**Figure 4:**
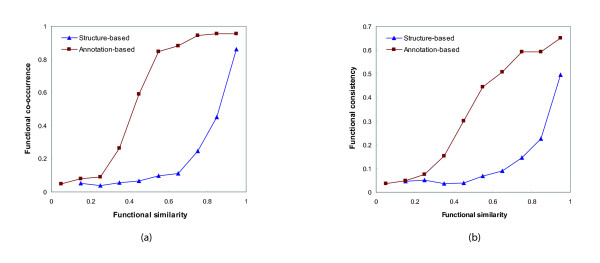
**(a) Functional co-occurrence and (b) functional consistency of interacting protein pairs with respect to their functional similarity**. We investigated the functional co-occurrence and functional consistency of the interacting protein pairs from DIP with respect to their functional similarity rates in the range between 0 and 1. The similarity was measured by Formula 13 and 14. As the similarity by both measurements becomes higher, the functional co-occurrence and consistency monotonically increase. However, the annotation-based similarity performs better than the structure-based similarity because there are not enough variations of functional co-occurrence and consistency up to 0.7 of the structure-based similarity. It indicates that the annotation-based method correctly quantified the functional similarity of interacting proteins.

We additionally investigated the appropriateness of functional similarity, which is scored as a real number between 0 and 1. We measured the functional similarity using Formula 13 and 14, and divided the interacting pairs into ten groups by the range of 0.1 of similarity. We then calculated the average functional co-occurrence and functional consistency for each group. The results are shown in Figure 4(a) and 4(b). Both functional co-occurrence and functional consistency monotonically increase as functional similarity becomes higher. In Figure 4(a), a small variation of functional co-occurrence is shown up to 0.7 of similarity by the structure-based method. However, when we used the annotation-based method, low co-occurrence rates are shown with below 0.3 of similarity and a rapid growth is appeared between 0.3 and 0.5 of similarity. The interacting protein pairs, whose similarity is greater than 0.7, have the co-occurrence rates that is higher than 0.94. However, according to the observation in Figure 1(b), the amount of them corresponds to less than 0.04% of the total number of interactions. In Figure 4(b), the growing patterns of functional consistency are similar to those of the functional co-occurrence. However, when we use the annotation-based method, functional consistency gradually increases across all the range of similarity. As a result from Figure 4, the annotation-based method can correctly quantify the functional similarity between interacting proteins.

### Cross-validation of function prediction

We assessed the performance of our function prediction approach by the leave-one-out cross-validation method. For each distinct protein in the actual annotations from FunCat in MIPS, we assumed it was un-annotated and predicted its functions. The prediction performance was evaluated using precision and recall (also called true positive rate). We transformed the format of the actual annotations, from the set of proteins annotated on each functional category to the set of functions for each protein. Let *M*_*i *_be the set of functions from the actual annotation in MIPS for a protein *p*_*i*_, *N*_*i *_be the set of functions predicted by our algorithm for *p*_*i*_, and *K*_*i *_be the set of common functions of *M*_*i *_and *N*_*i*_. Precision and recall are then described as:

(9) $$Precision=\frac{\sum_{i}^{n}\left|K_{i}\right|}{\sum_{i}^{n}\left|N_{i}\right|},$$

and

(10) $$Recall\;(True\;Positive\;Rate)=\frac{\sum_{i}^{n}\left|K_{i}\right|}{\sum_{i}^{n}\left|M_{i}\right|},$$

where |*K*_*i*_| is the size of the set *K*_*i *_and *n *is the total number of distinct proteins that are annotated on at least one functional category and have the interaction evidence.

**Figure 5:**
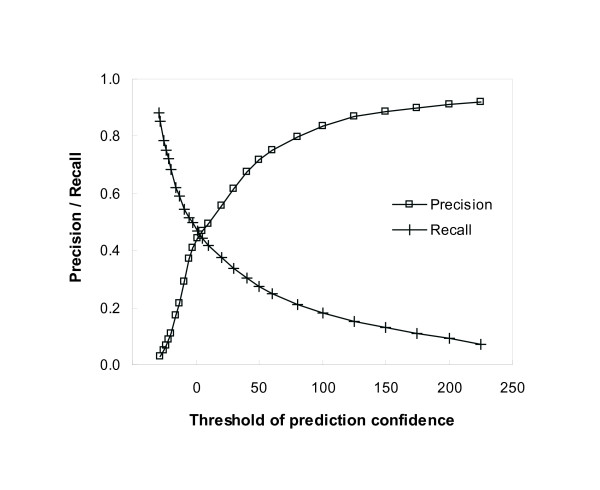
**Precision and recall plots by cross-validation for protein function prediction**. The performance of our function prediction algorithm was assessed by the leave-one-out cross-validation using the proteins that appear in the interaction data from DIP and are annotated on the functional categories in MIPS. As a higher threshold of prediction confidence is used, precision increases whereas recall decreases.

Figure 5 shows the precision and recall plots with respect to the threshold of prediction confidence, which is a user-dependent parameter in our algorithm. When we use 200 as the threshold of prediction confidence, our algorithm predicts no or a very few functions for each protein, but most of the functions are correctly predicted comparing to the actual annotations. It results in the precision of greater than 0.9. As a lower threshold is used, recall increases while precision decreases monotonically. Approximately, when the recall is 0.2 and 0.4, we had the precision of 0.8 and 0.5, respectively.

### Comparison of prediction performance

We compared the prediction performance with two previous methods, which are currently the most competing. One is the approach weighting the functional similarity of direct and indirect neighbors (level-2 neighborhood) [19], and the other is the prediction method based on the annotation patterns in the neighborhood [20]. The first method computes the likelihood that an unknown protein *p *has a function using the functional similarity weights between *p *and level-1 or level-2 neighbors. The functional similarity weight of two proteins is calculated by the commonality of their neighbors in the protein interaction network. Since we used the same interaction data from DIP as inputs for all the methods, we did not consider the reliability of the data source. We used a threshold of the likelihood to generate the output set of predicted functions for each protein. We then obtained different output sets by various thresholds. The sets of predicted functions become larger as the threshold lowers.

The second method constructs the set of annotation neighborhood patterns for each function, and computes the similarity between the annotation neighborhood pattern of an unknown protein and the set of annotation neighborhood patterns of each function. We used the annotations from the functional categories in MIPS since they are used as a ground truth in our experiment. We set the parameter *d *= 1 and did not consider the edge weights, i.e., assigned 1 to each edge weight. We used the similarity of annotation neighborhood patterns as a threshold.

**Figure 6:**
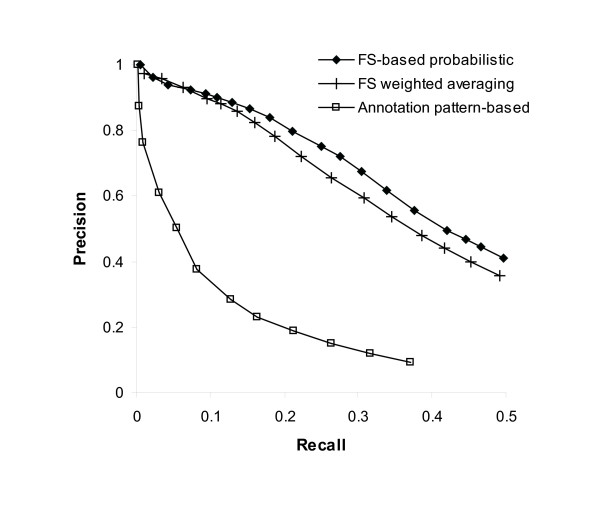
**Performance comparison of three function prediction methods**. The prediction performance by precision-recall of our functional similarity-based probabilistic approach was compared to that of two competing methods: the FS weighted averaging method and the pattern-based prediction method. The methods could predict the different number of functions for each protein with a selected threshold. Each method then generated several different output sets by varying the threshold. We calculated the precision and recall of each output set. Our approach remarkably outperforms the annotation pattern-based method and has higher precision than the FS weighted averaging method when the recall is greater than 0.07.

Figure 6 shows the relationships of precision and recall resulted from the three methods. Our functional similarity-based probabilistic approach remarkably outperforms the annotation pattern-based method. Because the annotation pattern-based method did not distinguish between general and specific functions, it could not predict general functions with higher confidence than specific functions. Thus, even though it precisely predicted the specific functions, the overall accuracy of the annotation pattern-based method was much lower than those of the other methods. Our probabilistic approach also has higher precision than the FS weighted method when the recall is greater than 0.07. When the recall is greater than 0.2, our approach has the precision of more than 0.05 points higher than the FS weighted method. This result indicates that integrating of protein interaction data with the annotations in GO explicitly improves the function prediction accuracy. Because the other two methods directly use the binary representation of connectivity from current interaction data, they are unable to overcome the critical problem of the interactions that are false positive or not related to functional connections, even though the FS weighted method may partly solve the false negative interaction problem.

In the experiment above, we implemented the function prediction algorithms using a threshold of prediction confidence. If a protein has low rates of prediction confidence for any function, the prediction result for the protein is not generated. For the comprehensive comparison of the prediction accuracy for all proteins, we implemented the algorithms again using a threshold of the number of predicted functions, *δ*. That is, for each protein, *δ *best predicted functions are generated as an output. As a set of functions to be predicted, we used the ones on the same level in a hierarchy. The previous experiment used all the functions in the hierarchical structure. However, predicting very general functions is meaningless when a small number of functions are predicted for each protein. We thus selected only the functional categories and their annotations on the third level from the top in the functional hierarchy. We then evaluated the prediction accuracy using precision, but if a protein had the functions less than *δ *in the actual annotations from MIPS, the number of annotated functions, *M*_*i*_, was used instead of the number of predicted functions, *N*_*i *_(equivalent to *δ*), in Formula 9. Table 1 shows the prediction accuracy of our functional similarity-based probabilistic approach comparing to the FS weighted averaging method, annotation pattern-based method and neighborhood-based chi-square method. Overall, our approach outperforms the others across any *δ *value up to 6. It means our approach predicts the specific functions of any protein with higher accuracy than the previous methods.

**Table 1:** Function prediction accuracy comparison

*δ*	1	2	3	4	5	6
functional similarity-based method	0.446	0.432	0.434	0.451	0.472	0.490
FS weighted averaging method	0.417	0.406	0.415	0.437	0.458	0.479
annotation pattern-based method	0.306	0.311	0.321	0.340	0.362	0.386
neighborhood-based chi-square method	0.294	0.302	0.319	0.343	0.370	0.398

The prediction accuracy (precision) of our functional similarity-based probabilistic approach was compared to three previous methods, the FS weighted averaging method, the annotation pattern-based method and the neighborhood-based chi-square method. The methods predicted the fixed number *δ *of functions for each protein. Our approach outperforms the others across any *δ *up to 6.

### Function prediction for unknown proteins

According to the most recent version of functional annotations in MIPS, a significant number of uncharacterized proteins in Saccharomyces cerevisiae still exist. We implemented our algorithm to predict their functions. Among the unknown proteins in MIPS, we selected only the proteins that have more than three interacting partners in DIP to additionally avoid the effect of false positive interactions. For each selected protein, our algorithm generated a list of functions with prediction confidence, which is the value of log(*λ*_*f*_) where *λ*_*f *_is calculated by Formula 8. A protein can thus correspond to more than one predicted function with different confidence rates. Table 2 shows the list of predicted functions when we use 32 as the threshold of prediction confidence and filter out excessively general functions, e.g., the categories on the first or second level in the functional hierarchy from MIPS. The functions of YJL058C and YGR163W were predicted with high confidence, greater than 100. These results suggest new functional annotations for currently unknown proteins. The whole prediction results with 10 as the threshold of prediction confidence are provided in the supplementary material [see Additional file 1].

**Table 2:** Function prediction results for unknown proteins

unknown	predicted function		confidence
		
	ID in MIPS	description	
YAL027W	02.16.01	alcohol fermentation	95.1
YAL053W	01.05	C-compound and carbohydrate metabolism	34.3
YAR027W	20.01.27	drug/toxin transport	66.0
YBL046W	01.02	nitrogen, sulfur or selenium metabolism	34.2
YBL046W	14.07.03	modification by phosphorylation/dephosphorylation	37.0
YCL028W	01.03.07	deoxyribonucleotide metabolism	62.7
YFL042C	02.16.01	alcohol fermentation	46.3
YGL230C	20.01.11	amine/polyamine transport	32.7
YGR163W	14.13.04	lysosomal and vacuolar protein degradation	59.9
YGR163W	20.01.01	ion transport	64.1
YGR163W	34.01.01	homeostasis of cations	115.2
YHL042W	14.07.02.01	glycosylation/deglycosylation	49.2
YHR105W	14.07.02.01	glycosylation/deglycosylation	49.2
YHR140W	20.01.27	drug/toxin transport	66.0
YJL058C	01.04	phosphate metabolism	36.0
YJL058C	01.06	lipid, fatty acid and isoprenoid metabolism	215.7
YJL058C	42.04	cytoskeleton/structural proteins	42.6
YJL122W	10.03.01.01	mitotic cell cycle	34.4
YLR376C	10.03.02	meiosis	36.6
YLR376C	10.03.04	nuclear or chromosomal cycle	37.1
YKL065C	20.01.11	amine/polyamine transport	50.3
YKL065C	32.05.01	resistance proteins	51.9
YPL264C	01.20.19.01	metabolism of porphyrins	54.9

For each unknown protein, our probabilistic approach predicted a set of functions with prediction confidence, log *λ*. The prediction results, whose confidence is greater than 32, were listed. A protein can have more than one function predicted with different confidence rates.

### Prediction of subcellular localization

Our probabilistic framework can be also applied to the prediction of subcellular localization. All implementation was the same to the function prediction process except the calculation of functional similarity. The functional similarity was measured with the GO terms from cellular component category in the GO database. We used total 556 GO terms and their annotations. Each interaction thus has different similarity from previous experiments. Comparing to the distribution of interacting pairs with respect to the functional similarity in Figure 1, much larger portion of them have low rates of similarity, which are less than 0.2. For each unknown protein, our algorithm generated a list of subcellular components with prediction confidence. A protein can thus correspond to more than one predicted subcellular component with different confidence rates. The localization prediction results are listed in Table 3 when we use 40 as the threshold of prediction confidence. The localization of YJR033C, YJR091C and YOR076C was predicted with very high confidence, greater than 200.

**Table 3:** Localization prediction results for unknown proteins

unknown	predicted subcellular localization		confidence
		
	ID in MIPS	description	
YER070W	755	mitochondria	90.7
YJR033C	750	nucleus	215.9
YJR091C	722	integral membrane/endomembranes	49.6
YJR091C	725	cytoplasm	213.2
YJR091C	770	vacuole	52.1
YLL038C	705	bud	50.0
YML023C	722	integral membrane/endomembranes	119.8
YML023C	750	nucleus	191.3
YNL293W	705	bud	81.0
YNL293W	715	cell periphery	69.6
YNL293W	730	cytoskeleton	54.3
YOR076C	750.05	nucleolus	215.8
YOR076C	755	mitochondria	60.3
YOR231W	705	bud	168.7
YOR231W	715	cell periphery	58.0
YOR231W	730	cytoskeleton	45.3
YOR231W	750	nucleus	88.0

For each unknown protein, our probabilistic approach predicted a set of subcellular components with the prediction confidence, log *λ*. The prediction results, whose confidence is greater than 40, were listed. A protein can have more than one subcellular component predicted with different confidence rates.

## Discussion

Through recent advances of high-throughput techniques, a significant amount of protein-protein interaction data have been accumulated. Protein function has been predicted from the interaction data because the evidence of interaction can be interpreted as functional links. However, we observed only a small fraction of current interaction data from major interaction databases are related to functional linkage. The results in Figure 1 indicate that more than or around 60% of interacting protein pairs are not linked by similar functions. In other words, at most 40% of protein pairs have been motivated by similar functions. This rate goes beyond the potential false positives of experimentally determined interactions, claimed in [34]. This observation has been also demonstrated by the limited accuracy of previous function prediction methods, which are based on the connectivity of protein interaction networks, as shown in Figure 6 and Table 1.

Our function prediction algorithm uses a probabilistic formula derived from functional similarity between proteins that interact with each other. The functional similarity can be measured using the structure or annotations from Gene Ontology (GO). Various measurements for the functional similarity were evaluated in terms of functional co-occurrence and consistency of interacting pairs. The experimental results in Figure 2, 3 and 4 show that the annotation-based method performs the assessment of similarity better than the structure-based method. It indicates that the current GO structure itself is an imperfect resource to identify functional linkage. In our experiments, function prediction has been conducted with yeast protein-protein interaction data. However, our probabilistic framework can be well-applicable to higher-level organisms because of its efficiency.

## Conclusion

Functional characterization of proteome is a central goal in the field of Bioinformatics. The experimentally determined protein-protein interactions are crucial data sources to uncover the functional knowledge of uncharacterized proteins. However, a pre-process to assess the functional linkage of interacting proteins from current interaction data is required for predicting protein function successfully.

In this article, we presented a novel concept for integrating the connectivity of protein interaction networks with already published annotation data in Gene Ontology (GO). Our results imply that function prediction from protein interaction networks has been calibrated by integrating with the functional knowledge from GO. Clearly, the prediction accuracy can be more improved by the integration of multiple data sources available, which are relevant to functional linkage. Furthermore, developing effective integration models for the explosive amount of heterogeneous biological data sources is promising in future research for functional knowledge discovery.

## Methods

### Structure-based functional similarity measures

To measure the functional similarity of an interacting protein pair, we can use the path length between two GO terms in the GO structure. We first find the most specific GO terms on which each interacting protein is annotated, and then selects the term pair that has the shortest path between the terms. The shortest path length can be scaled down by the maximum depth of GO and applied log smoothing.

(11) $$sim(p_{1},p_{2})=-\log\left(\frac{\min_{i,j}length(T_{i},T_{j})}{2\times depth}\right),$$

where *T*_*i *_and *T*_*j *_are the GO terms whose annotations include the interacting proteins *p*_1 _and *p*_2_, respectively, and *depth *denotes the maximum path length from the root term to a leaf.

As an alternative way, we can consider the proportion of common parent GO terms. A set of parent terms of a term *T*_*i *_represents all the terms on the paths towards the root from *T*_*i*_in the DAG structure. *T*_*i *_is exclusive and the root term is inclusive. The similarity is then calculated by the common parent GO terms over all distinct parent GO terms.

(12) $$sim(p_{1},p_{2})=\underset{i,j}{\max}\left(\frac{P_{T_{i}}\cap P_{T_{j}}}{P_{T_{i}}\cup P_{T_{j}}}\right),$$

where $P_{T_{i}}$ and $P_{T_{j}}$ is the set of GO parent terms of *T*_*i *_and *T*_*j*_, respectively.

**Figure 7:**
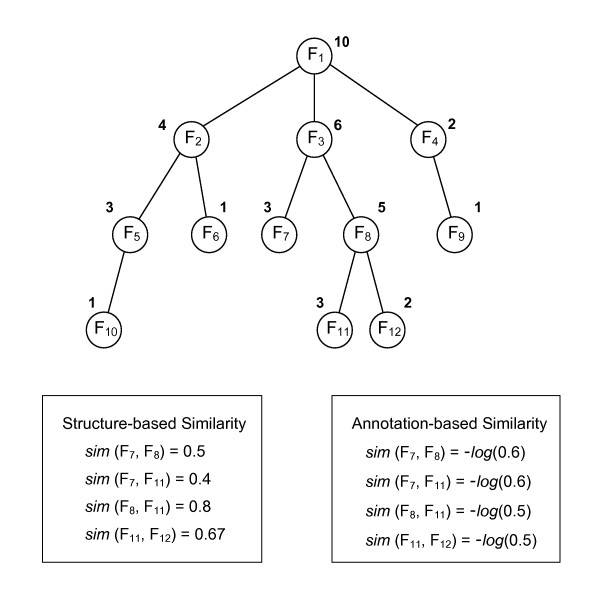
**Examples of structure-based similarity and annotation-based similarity between functions in a hierarchy**. Each circle represents a function, and each edge is a general-to-specific relationship between two functions. The depth of a function is the path length from the root to the function. The number close to each function represents the number of proteins annotated on the function. The structure-based similarity between two functions is calculated by the ratio of the depth of the most specific common function to the average depth of the functions of interest (Formula 13). The annotation-based similarity between two functions becomes the negative logarithm of the proportion of proteins annotated on the most specific common function (Formula 14). Some examples of the structure-based and the annotation-based similarity between two functions in the hierarchy are shown in the boxes.

As a combination of the two factors, path length and common parent terms, we can use the maximum path length from the root to the most specific common term on which the interacting proteins are annotated together. It is then normalized by the path lengths from the root to the most specific terms which have the annotation of each interacting protein.

(13) $$sim(p_{1},p_{2})=\underset{i,j}{\max}\left(\frac{2\times length(T_{r},T_{c})}{length(T_{r},T_{i})+length(T_{r},T_{j})}\right),$$

where *T*_*r *_is the root term and *T*_*c *_is the most specific common parent term of *T*_*i *_and *T*_*j*_. Figure 7 shows the examples of the structure-based similarity by Formula 13. The similarity between a parent and a child is higher than that of siblings, and the similarity of siblings on a lower level is higher than that of siblings on a higher level in the hierarchy. The functional similarity of two proteins then becomes the maximum similarity of the pair-wise functions they have.

### Annotation-based functional similarity measures

In Information Theory, self-information is a measure of the information content associated with the outcome of a random variable. The amount of self-information contained in a probabilistic event *c *depends on the probability *P*(*c*) of the event. Specifically, the smaller the probability of the event, the larger the self-information associated with receiving information when the event indeed occurs. The information content of a concept *C *is defined as the negative log likelihood of *C*, -log *P*(*C*). The similarity between two concepts is measured by their commonality, i.e., the information content of the most specific common concept [35].

In the same manner, the information content of a GO term *T *can be represented as -log *P*(*T*) where *P*(*T*) is the proportion of the proteins annotated on *T*, and the similarity of two terms *T*_*i *_and *T*_*j *_is calculated by -log *P*(*T*_*k*_) where *T*_*k *_is the most specific common term of *T*_*i *_and *T*_*j*_. To measure the functional similarity of interacting proteins *p*_1 _and *p*_2_, we list all pair-wise terms that have the annotations of *p*_1 _and *p*_2 _respectively and select the maximum similarity of the pairs. Suppose *S*_*k*_(*p*_1_,*p*_2_) is a set of proteins annotated on the GO term *T*_*k *_that includes both *p*_1 _and *p*_2 _in the annotation. The functional similarity between *p*_1 _and *p*_2 _is defined as:

(14) $$sim(p_{1},p_{2})=-\log\left(\underset{k}{\min}P(T_{k})\right)=-\log\left(\underset{k}{\min}\frac{\left|S_{k}(p_{1},p_{2})\right|}{\left|S_{root}\right|}\right).$$

Figure 7 shows the examples of the annotation-based similarity by Formula 14. The functional similarity can be normalized by the number of annotated proteins on the individual terms, *T*_*i *_and *T*_*j*_.

(15) $$sim(p_{1},p_{2})=\underset{i,j,k}{\max}\left(\frac{2\cdot \log P(T_{k})}{\log P(T_{i})+\log P(T_{j})}\right).$$

## Authors' contributions

YRC designed and implemented the data integration and prediction algorithm, analyzed the results and drafted the manuscript. LS partly analyzed the results. MR and AZ coordinated the project and revised the manuscript. All authors read and approved the final manuscript.

## Supplementary Material

Additional file 1 **Function prediction results for unknown proteins.** For each unknown protein, our probabilistic approach predicted a set of functions with prediction confidence of greater than 10. The predicted functions are described as the IDs used in MIPS. Click here for file
