# Supplementary material for: A probabilistic framework to predict protein function from interaction data integrated with semantic knowledge
Source: BMC Bioinformatics. 2008 Sep 18;9:382. doi: 10.1186/1471-2105-9-382 (PMC2570367; doi:10.1186/1471-2105-9-382)
Supplement: Additional file 1 — Function prediction results for unknown proteins. For each unknown protein, our probabilistic approach predicted a set of functions with prediction confidence of greater than 10. The predicted functions are described as the IDs used in MIPS. [file 1471-2105-9-382-S1.pdf]

unknown	predicted function ID in MIPS (prediction confidence)									
YAL018C	20.01.11	(15.06)	20.01.27	(20.57)						
YAL027W	02.16.01	(95.10)								
YAL034C	02.16.01	(46.30)								
YAL053W	1	(37.12)	1.05	(34.31)	2	(57.15)	20	(21.24)	42	(27.75)
	42.01	(42.33)								
YAR027W	01.20.19	(26.53)	01.20.19.01	(26.14)	20.01.27	(65.96)	20.03.01	(26.70)		
YBL046W	1	(62.95)	1.02	(34.22)	1.04	(20.42)	1.05	(27.62)	14	(34.84)
	14.07	(42.21)	14.07.03	(36.97)	40	(14.77)	40.01	(16.05)		
YBR197C	01.03.16.03	(15.85)								
YBR270C	20.03.01	(10.61)								
YBR280C	01.03.01	(12.66)								
YCL028W	01.03.07	(62.67)	18.02.01.02	(15.85)	30.01.09.11	(14.98)	40.01.05	(17.55)		
YCR007C	01.03.16.03	(15.85)								
YCR072C	1	(24.02)	1.04	(16.48)	11	(73.22)	11.04	(131.94)	11.04.01	(77.30)
	12	(125.32)	12.01	(53.15)	12.01.01	(26.58)	14	(28.73)	16.19	(10.87)
	16.19.03	(11.11)	42	(17.25)	42.04	(17.00)				
YDL089W	32.07.07	(15.60)								
YDL204W	30.01.09.11	(14.98)								
YDL233W	20.01.27	(20.57)								
YDR084C	01.03.01	(12.66)								
YDR128W	20.01.11	(15.06)								
YEL041W	1	(89.60)	1.03	(120.96)	1.04	(56.52)	1.07	(215.58)	34	(68.72)
	34.01	(177.81)	34.01.01	(153.82)	34.01.01.01	(137.10)				
YER067W	32.05	(22.06)	32.05.01	(24.83)						
YER071C	01.20.19	(26.53)	01.20.19.01	(26.14)						
YER113C	20.01.27	(20.57)								
YFL042C	02.16.01	(46.30)								
YFL049W	1	(11.54)	11	(13.52)						
YFL062W	20.03.01	(10.61)								
YGL230C	20.01.11	(32.66)								
YGL250W	40.01.05	(17.55)								
YGL259W	01.03.01	(12.66)								
YGR017W	40.01.05	(17.55)								
YGR111W	20	(19.17)	20.09	(10.85)						
YGR163W	1	(28.18)	14	(54.97)	14.13	(19.02)	14.13.04	(59.94)	20	(60.31)
	20.01	(63.35)	20.01.01	(64.10)	34	(48.27)	34.01	(130.01)	34.01.01	(115.24)
YGR295C	20.03.01	(10.61)								
YHL042W	01.20.19	(26.53)	01.20.19.01	(26.14)	14.07.02.01	(49.18)				
YHR105W	14.07.02.01	(49.18)								

YHR140W	01.20.19	(26.53)	01.20.19.01	(26.14)	20.01.01.01.01.01	(11.27)	20.01.11	(32.66)	20.01.27	(65.96)
	20.03.01	(23.70)	32.05	(22.06)	32.05.01	(24.83)	32.05.01.03	(31.65)		
YHR177W	20.01.27	(20.57)								
YHR199C	32.05	(22.06)	32.05.01	(24.83)	32.05.01.03	(31.65)	32.07.07	(15.60)		
YIL023C	32.07.07	(15.60)								
YJL057C	32.05	(22.06)	32.05.01	(24.83)	32.05.01.03	(31.65)				
YJL058C	1	(111.82)	1.04	(36.01)	1.06	(215.69)	10	(16.47)	12	(53.24)
	16	(64.92)	16.01	(13.92)	20	(71.04)	20.09.07	(33.33)	30	(26.78)
	30.01.05	(37.61)	30.01.05.05	(14.33)	30.01.05.05.01	(11.73)	40	(24.38)	40.01	(26.27)
	42	(33.41)	42.04	(42.60)						
YJL122W	10	(32.13)	10.03	(20.69)	10.03.01	(21.41)	10.03.01.01	(34.44)	42	(30.57)
YKL065C	01.20.19	(26.53)	01.20.19.01	(26.14)	20.01.01.01.01.01	(11.27)	20.01.11	(50.25)	20.01.27	(65.96)
	20.03.01	(10.61)	30.01.09.11	(14.98)	32.05	(46.30)	32.05.01	(51.89)	32.05.01.03	(65.58)
YKL183W	01.03.16.03	(15.85)								
YLL023C	01.03.16.03	(15.85)	20.01.27	(20.57)						
YLR125W	01.03.16.03	(15.85)								
YLR241W	20.01.01.01.01.01	(11.27)	20.01.27	(20.57)						
YLR254C	10	(13.21)	10.03.04	(26.14)						
YLR297W	20.01.11	(15.06)	20.01.27	(20.57)						
YLR376C	10	(59.40)	10.03	(37.56)	10.03.02	(36.61)	10.03.04	(37.10)	10.03.04.05	(25.74)
YLR446W	32.05	(22.06)	32.05.01	(24.83)	32.05.01.03	(31.65)				
YML011C	01.03.01	(12.66)								
YMR206W	01.20.19	(26.53)	01.20.19.01	(26.14)						
YNL092W	32.05	(22.06)	32.05.01	(24.83)	32.05.01.03	(31.65)				
YNL095C	20.01.27	(20.57)	32.05	(22.06)	32.05.01	(24.83)	32.05.01.03	(31.65)		
YNL176C	18.02.01.02	(15.85)								
YNL260C	20.03.01	(10.61)								
YNR068C	20.01.27	(20.57)								
YOL087C	20.03.01	(10.61)								
YOL131W	01.03.16.03	(15.85)								
YOR104W	01.03.16.03	(15.85)								
YOR161C	01.20.19	(26.53)	01.20.19.01	(26.14)	20	(40.51)	20.01	(37.63)	20.01.01.01.01.01	(11.27)
	20.01.07	(18.83)	32.05	(22.06)	32.05.01	(24.83)	32.05.01.03	(31.65)		
YOR164C	34.11.03.05	(19.49)								
YOR220W	32.05	(22.06)	32.05.01	(24.83)	32.05.01.03	(31.65)				
YOR315W	16	(10.48)	30	(18.23)	30.01	(15.66)	30.01.05	(10.13)	42	(17.06)
	42.04	(16.80)	43.01.03.05	(11.18)						
YPL264C	01.03.01	(12.66)	01.20.19	(55.50)	01.20.19.01	(54.85)	20.01.01.01.01.01	(11.27)	32.05	(22.06)
	32.05.01	(24.83)	32.05.01.03	(31.65)						
YPR084W	01.03.01	(12.66)								
YPR085C	01.03.01	(12.66)								
